# Acceptability of a balanced energy protein (BEP) supplement for pregnant women in Bangladesh

**DOI:** 10.1111/mcn.13587

**Published:** 2023-11-22

**Authors:** Eleonor Zavala, Atiya Rahman, Anna Kalbarczyk, Mary de Boer, Nazrana Khaled, Barnali Chakraborty, Hafizur Rahman, Hasmot Ali, Rezwanul Haque, Kaniz Ayesha, Towfida J. Siddiqua, Kaosar Afsana, Parul Christian, Andrew L. Thorne‐Lyman

**Affiliations:** ^1^ Department of International Health Bloomberg School of Public Health, Johns Hopkins University Baltimore Maryland USA; ^2^ Humanitarian Hub James P. Grant School of Public Health, BRAC University Dhaka Bangladesh; ^3^ The JiVitA Maternal and Child Health and Nutrition Research Project Rangpur Bangladesh

**Keywords:** acceptability, adherence, balanced energy protein supplement, Bangladesh, mixed methods, pregnancy nutrition, pregnant women

## Abstract

Balanced energy protein (BEP) supplementation in pregnancy is recommended in the context of undernutrition for the reduction of small‐for‐gestational age neonates and stillbirths. To inform an effectiveness trial, we evaluated the acceptability of a packaged, ready‐to‐eat fortified BEP product among women of reproductive age and their health care providers (HCPs) in rural Bangladesh and explored the feasibility of adhering to daily supplementation. We implemented a formative study using focus groups discussions with women (*n* = 29) and HCPs (*n* = 17) to introduce the product and investigate components of acceptability. A “trials of improved practice” activity was conducted in subset of women (*n* = 16) to evaluate adherence to BEP over a 2‐week period, followed by focus group discussions to identify challenges with adherence and strategies employed. Contributors to BEP acceptability included the product's sensory attributes, such as taste, smell and texture; the attractive packaging and informative labelling; and the perceived benefits of use. Participants also identified household and community level factors influencing the adoption of BEP, such as trust in the provider, cultural beliefs on supplement use in pregnancy, and family member tasting and approval. Over the 2‐week period, women consumed over 80% of the supplements provided to them and identified strategies for adherence, including visual aids and reminders from family members or providers. HCPs recommended targeted communication messages for mothers‐in‐law to foster a supportive home environment. Findings informed changes to the BEP product to improve acceptability and shaped the content of communication messages to optimise adherence in a forthcoming effectiveness trial.

## Key messages

- The balanced energy protein (BEP) product introduced in this formative study was well accepted by women of reproductive age and their health care providers (HCPs), especially with regards to the overall taste, product packaging and labelling, and perceived benefits. Acceptability may be influenced by cultural beliefs around nutritional supplement use during pregnancy, and factors such as family members' approval of the product, and trust in the provider of BEP supplements.
- Women who consumed the product over 2 weeks had high daily adherence and identified strategies such as reminders from family members and/or providers and visual aids in the home to enhance regular use. HCPs recommended communication messages that encouraged buy in from husbands and mothers‐in‐law.
- Findings from this study informed changes to the BEP product format to improve acceptability and shaped the content of communication messages to encourage adherence in a forthcoming effectiveness trial.

## 1 INTRODUCTION

Maternal undernutrition in many low‐middle income settings is a strong determinant of poor health during pregnancy and adverse birth outcomes such as low birth weight (LBW). In national contexts of high undernutrition among women of reproductive age (WRA), the World Health Organisation recommends the use of balanced energy protein (BEP) supplements in pregnancy to reduce the risk of stillbirth and small‐for‐gestational age (SGA) neonates (World Health Organisation, 2016). In a meta‐analysis, BEP reduced the risk of SGA and stillbirth by 21% and 40%, respectively, and resulted in a mean 41 g increase in birth weight (Ota et al., 2015). Despite the evidence and recommendation, few countries have implemented national policies and programmes for BEP supplementation in pregnancy due, in part, to a lack of data on acceptability of available BEP formulations, and evidence of successful programme implementation.

Bangladesh is one of the few countries with a history of implementing large‐scale BEP supplementation for women in pregnancy. The Bangladesh Integrated Nutrition Programme, the first large‐scale government nutrition intervention in Bangladesh (1995–2002), provided low body mass index (BMI) (<18.5 kg/m^2^) pregnant women with “Pushti packets”, locally mixed, unfortified food supplements (Nahar et al., 2009). Evaluations of the programme were mixed: improvements were seen in maternal and child nutritional status and birth weight in intervention areas, but similar improvements were also seen in non‐intervention areas (The World Bank Office, Dhaka, 2005). Low enrolment, poor targeting, adherence challenges, and food sharing were identified as potential contributors to the lack of supplementation effect on pregnancy weight gain or birth weight (Khan et al., 2005). Current national nutrition programmes in Bangladesh do not include BEP, though locally produced, ready‐to‐use therapeutic foods (RUTFs) are in use in Rohingya refugee camps, including for pregnant women (World Food Programme, 2021).

Despite recent improvements in the national rate of undernutrition, Bangladesh continues to have high levels of maternal undernutrition and poor birth outcomes, particularly at sub‐national levels and among poorer families (National Institute of Population Research and Training NIPORT & ICF, 2020). Given the nutritional burden in Bangladesh and the strong political momentum to address the inequities in maternal and child undernutrition, BEP supplementation is an intervention with great promise, particularly since linear growth at birth strongly predicts linear growth and stunting at 24 months (Krebs et al., 2022). Given the costs of this intervention, targeted provision of BEP could have advantages including higher impact and cost‐effectiveness (Christian et al., 2020). Additionally, given that rates of overweight and obesity are rapidly rising in many low‐ and middle‐income countries, targeting could help to avert provision of excess calories to individuals that may not benefit from the supplement, possibly avoiding adverse outcomes. Our group is undertaking an effectiveness study in northwestern Bangladesh to test targeting as an approach for BEP supplementation during pregnancy. A previous nutrition intervention trial conducted in this area between 2008 and 2012 found 40.1% of women entering pregnancy had a BMI less than 18.5 kg/m^2^, and thus falling within the WHO's contextual recommendation for BEP supplementation (West et al., 2014). In preparation for the study and to inform its implementation, we undertook a comprehensive formative study using qualitative methods and a locally manufactured prototype BEP product. The BEP product composition aligns with recommendations from an expert consultation on the nutritional composition needed to meet the dietary requirements of pregnant women (Members of an Expert Consultation on Nutritious Food Supplements for Pregnant and Lactating Women, 2019).

The aims of this formative research study included: (1) understanding the perceptions and acceptability of this BEP product among WRA and their health care providers (HCPs); and (2) to explore the feasibility of adhering to daily supplementation over a short period and identifying potential strategies for adherence support.

## 2 METHODS

### 2.1 Setting

The formative research was conducted in three unions of the Gaibandha district of northwestern Bangladesh in the JiVitA study area. JiVitA is a maternal and child health and nutrition project established in 2000, serving a predominantly rural area and agrarian society. Previous studies in this area have revealed a high burden of low BMI and micronutrient deficiencies among pregnant women, and high rates of adverse birth outcomes, including 45.7% LBW among live‐births (West et al., 2014).

### 2.2 Intervention

**Figure 1:**
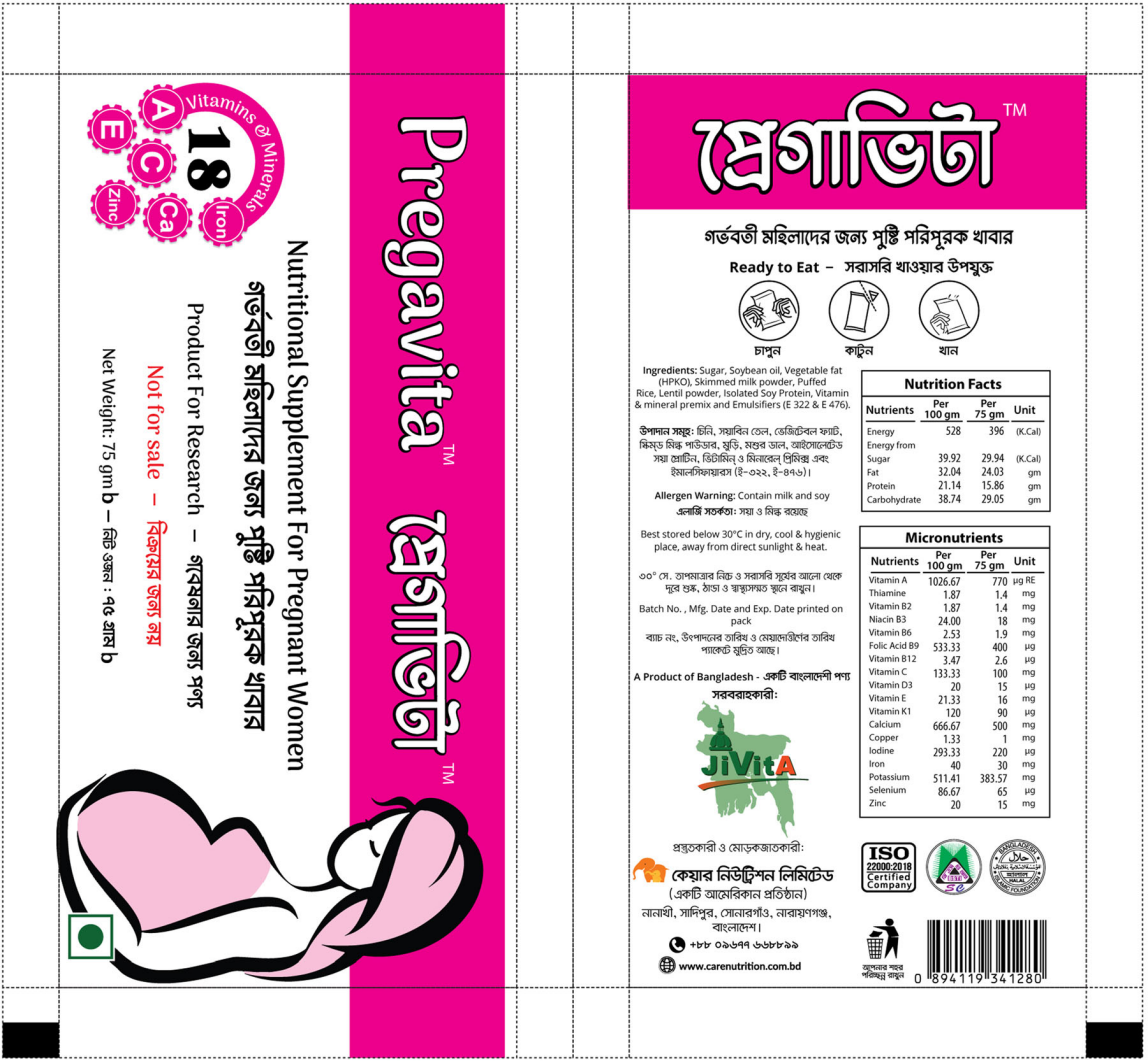
Pregavita BEP supplement packet. Legend: Label design for the BEP product used in the study, including nutritional facts and pictorial representation of the target recipient (pregnant woman). BEP, balanced energy protein.

The BEP supplement, developed by Frontier Nutrition in Bangladesh, was a lipid‐based, ready‐to‐use paste made with puffed rice, lentil powder, milk solids and vegetable oils packaged in a 75 g daily serving sachet. The product was shelf‐stable up to 12 months and was designed to be consumed immediately after opening, and women were advised to throw away any opened and partially consumed sachets at the end of the day. Figure 1 illustrates the packaging of the product which included nutrition facts, easy to read instructions, and pictorial aids.

### 2.3 Study design and tools

Mixed methods were used to address study aims. Perceptions about the product were explored using focus group discussions (FGDs) with WRA and HCPs. We conducted five FGDs with 5–7 WRAs (*n* = 29), and three FGDs with 5–6 HCPs (*n* = 17). Semi‐structured focus group guides containing open‐ended questions were developed to capture attitudes about diet in pregnancy and existing nutritional products for pregnant women, and to introduce the BEP product and explore different components of acceptability (Supporting Information). We evaluated multiple dimensions of acceptability including sensory attributes (taste, smell, texture); visual characteristics of the packaging; frequency and quantity of consumed portions; and perceived benefits and/or harms of use. Samples of the product were given to each participant to observe and taste, followed by a guided discussion to evaluate acceptability of supplementation from the potential beneficiary and provider perspectives.

We drew on the approach of trials of improved practice (TIPS) developed by the Manoff Group (Manoff Group, 2006), to explore the practice of daily BEP consumption over a 2‐week period and identify strategies for adherence. Two weeks was considered an appropriate time period to test adherence and the palatability of the product and to understand the possibility of sharing in the home. To rapidly inform implementation strategies related to the BEP intervention before the start of the trial and to reduce the risk of COVID transmission at the time of data collection, a sub‐sample of three groups of WRA (*n* = 16) were randomly selected to participate in the TIPS activity. They were provided 14 BEP packets and given relevant messages, including ways to consume BEP daily (between meals or throughout the day), instructions to not share with others, and nutritional value of the supplement. A brief, quantitative survey was administered by phone at 8 and 15 days to capture self‐reported consumption and sharing practices. Focus groups were reconvened after the trial period to reflect on their experiences with daily consumption, including ease and frequency of use, descriptions of sharing, and strategies for adherence (Supporting Information).

### 2.4 Recruitment, sampling and data collection

Participants were identified by project hired community health research workers (CHRWs), who drew up lists of married women in their communities who were pregnant or had recently given birth, given their ability to reflect on possible perceptions spanning the entirety of pregnancy. The CHRWs visited these women in their homes and administered a general recruitment script to inform participants on the study aims and asked screening questions to collect information on age, parity, education, and socioeconomic status. We then applied a purposive, criteria‐based selection technique to develop sampling frames that would ensure diversity and variability among respondents across the three unions. Selected women were subsequently approached by trained interviewers and administered an oral consent script. Local community health care workers engaged in antenatal and reproductive health care were purposely selected and included NGO health care workers from BRAC and government‐employed female welfare assistants (FWA) or female welfare visitors (FWV). They were recruited in local health centres in all three unions, and permission was obtained by the Ministry of Health and Family Welfare and BRAC.

Data was collected by three trained interviewers with degrees in anthropology and previous experience conducting qualitative data collection, along with two supervisors from JPGSPH (NK, AR) from January to March 2022. A 2‐week training involved qualitative research techniques, field guide pre‐testing, and human subjects research ethics. Scheduling of recruited participants was conducted by field supervisors, and the interviewers conducted the oral consent procedure before each FGD. FGDs were conducted in local field offices in the women's communities, in private rooms without interruption from community members. Each session lasted approximately 60–90 min. Data was audio recorded, transcribed into Bangla, and then translated into English. Data was stored on encrypted servers and deidentified before analysis.

### 2.5 Data management and analysis

We managed and analyzed data using Dedoose software. We used two analytical frameworks to build our initial code list (Pelto & Armar‐Klemesu, 2015; Tumilowicz et al., 2019). The implementation science in nutrition framework (Tumilowicz et al., 2019) informed our five domains of interest and the cultural‐ecological model of food and nutrition (Pelto & Armar‐Klemesu, 2015) helped us predefine codes at the individual, household, and community levels. Three members from JPGSPH and three members of BSPH conducted the coding. After coding 10% of the transcripts, we discussed and refined the code list to capture emerging themes. We conducted an inter‐rater reliability exercise to identify any differences in coding among team members and developed best practices to align coding. We then finalised the code list (Supporting information) and coded all remaining transcripts. For this analysis, we explored the following codes: adherence, taste/smell, texture, amount/supply, labelling/packaging, portion size, sensations/discomforts, sharing, storage; and where “BEP” co‐occurred with the following codes: cultural and social beliefs, access to resources, family support, household norms, acceptability, and healthy pregnancy. Organisation of quotes into thematic groups resulted in the emergence of important sub‐themes not fully captured in the initial code list. We applied the consolidated criteria for reporting qualitative research (COREQ) checklist to report the study procedures and findings (Supporting information) (Tong et al., 2007).

### 2.6 Reflexivity

Of the six authors who conducted the coding and analysis, five identify as female and one as male, and half are Bangladeshi nationals. Three have doctoral degrees and three have master's degrees in the field of public health or anthropology, all with previous experience collecting and analyzing qualitative data. Our previous involvements with qualitative research and the diversity of our group, both in background and in expertise, allowed us to question our interpretations and consider alternative perspectives throughout data collection and analysis. Although no authors are ‘insiders’ within the community of participants, we aimed to reduce biases by working closely with the interviewers, seeking clarification from community members when local terms and concepts arose, and by drawing on existing frameworks in the field of public health nutrition.

### 2.7 Ethical approval

This study was approved by the BRAC University James P. Grant School of Public Health and the Johns Hopkins University Bloomberg School of Public Health Institutional Review Boards.

## 3 RESULTS

### 3.1 Characteristics of study participants

Table 1 summarises participant characteristics in each focus group. Women were between 16 and 35 years of age and ranged in years of education. Most were non‐pregnant, and all but two had already had one child and each group contained women who had recently given birth. Of the 16 women in the TIPS groups, two were pregnant, and five were lactating (Table 1). Two HCP FGDs were conducted with BRAC health care workers in their mid‐thirties, the majority of which had a high school degree or higher. The third HCP FGD was conducted with FWAs and FWVs who were, on average, older and more experienced, but with similar levels of education.

**Table 1:** FGD participant characteristics.

**Health care providers**							
Group ID	Location	Number of participants	Type of providers	Place of work	Age^a^	Education level	Years of experience^a^
FGD_HCP_01	BMN	5	BRAC HCWs	Community	36 (33–40)	High school completed	15 (12–17)
FGD_HCP_02	SNR	6	BRAC HCWs	Community	34 (26–50)	Class 6 to 10 completed	9 (4–15)
FGD_HCP_03	SNR	6	FWA/FWV	Community	48 (26–54)	High school completed	27 (3–33)

Married women of reproductive age							
Group ID	Location	Number of participants	Age^a^	Number of children^a^	Pregnancy status	Education level	Occupation
MWRA_FGD_01	SRB	7	26 (16–35)	2 (0–3)	Non‐pregnant; 1 lactating	0 to masters level	None
MWRA_FGD_02	SRB	6	25 (19–34)	2 (1–4)	Non‐pregnant; 3 lactating	Class 2 to masters level	None
MWRA_TIPS_01	SNR	5	29 (26–31)	2 (2–4)	Non‐pregnant; 2 lactating	Class 5 to university	2 business owners
MWRA_TIPS_02	SRB	6	19 (18–20)	All had one child	Non‐pregnant; 1 lactating	Class 5 to 12	None
MWRA_TIPS_03	SRB	5	18 (16–21)	1 (0–1)	2 pregnant; 2 lactating; 1 NPNL	Class 7 to university	None

Abbreviation: FGD, focus group discussions.

^a^ Age, years of experience, and number of children presented as a mean with range across included participants.

We identified three major themes related to the acceptability of BEP supplementation among WRA: (1) Properties of the BEP product; (2) Household and community factors; and (3) Utilisation and adherence strategies.

### 3.2 BEP product properties

The BEP product was generally well‐liked by women and health providers. Participants reported enjoying the taste and appreciated the informative packaging. However, participants had mixed opinions about the smell and texture of the product.

#### 3.2.1 Sensory attributes

Women had a positive reaction after tasting the BEP and described it by comparing it to biscuits, powdered milk, and candy. While some women recommended the product could be made slightly sweeter, most described it as well balanced, as captured by one participant: “It tastes a little sweet and a little salty. It felt good to eat” (MWRA_TIPS_03). HCPs similarly expressed positive feedback when they tasted the product and felt the taste would be acceptable to pregnant women. One BRAC worker suggested the product could be prepared with different flavour profiles: “There will be no problem in eating this. If there was something ‘tok tok’ (sour) in this, then it would be tastier… It would be better if it were ‘jhal, mishty, tok tok’ (spicy, sweet, and sour)” (HCP_FGD_01).

While the taste of the BEP was well received, women had mixed perceptions regarding the smell. Some liked the smell, as described by a woman in the follow‐up TIPS discussion: “We like the smell of the food, this is just right, there is no need to change this” (MWRA_TIPS_02), but a couple women identified a lentil smell that they found off‐putting: “To me, the smell of the lentils was a bit too much, I felt. I couldn't eat the whole packet the first time. The pea/lentil that is included as one of the ingredients of the packet is smelly for me” (MWRA_TIPS_01). No other aversions to the smell of the product were described, though one woman suggested that the smell of the BEP could be less acceptable during the first trimester of pregnancy, when women may experience morning sickness and sensitivity to certain smells: “Those mothers who are in their first pregnancy, those who are two or three months into their pregnancy, they may have some problems. Someone may smell a bad smell… They can't eat, some can't tolerate the smell of milk, some can. But after three months of gestation, everyone can consume. There will be no problem” (MWRA_TIPS_01). All three HCP focus groups described the smell of the product as fine or acceptable.

Reactions to the texture of the lipid‐based paste were mixed, with some women describing it as “not too thick, not too thin” (MWRA_TIPS_02), whereas others explained the texture as ‘*ghono*’ (thick), ‘*atha atha*’ (sticky), and ‘*garo*’ (concentrated). One woman recommended thinning the product for a better mouth‐feel: “It would be better if the concentration of the food is a little lighter. After eating it, it sticks like glue in the mouth (‘*muker bhitor baje*’) so you need to suck on it slowly like chocolate or candy” (MWRA_TIPS_01). In one focus group, a woman described how the paste could be eaten with a spoon if made slightly thinner, but another woman responded that, “the packet can be cut on one side, and eaten like one eats ‘*achar*’ (pickles)” (MWRA_FGD_02). HCPs similarly expressed that the texture was either fine or could be diluted slightly. One health care worker suggested a powdered format, as found commercially with products like Mother Horlicks, could be an alternative to the paste: “There is Mother Horlicks as well, apa. That also has a system where you have to mix it with hot water; that's the instruction for eating it. I think it may have been better if this were like that too. You can make it a powder form” (HCP_FGD_01). However, a powdered format or other alternatives were not commonly suggested by women.

#### 3.2.2 Labelling and packaging

Participants described the labelling and packaging of the BEP as visually appealing and informative in identifying the target audience and nutritional content (Figure 1). When asked about the packaging, a woman responded, “The food packet is beautiful. Everything can be understood. This is nutritious for pregnant women” (MWRA_TIPS_02). One pregnant woman described how she identified with the image of the pregnant woman on the packet itself: “For example, this picture indicates that it is for pregnant women. Just like me, I am also pregnant. If I eat [BEP], it would be helpful for me” (MWRA_TIPS_03). Some women highlighted components of the ingredient list and provided their approval, “it is written on the packet which nutritious elements are in it, the ingredients used in making the foods, everybody will know that there is nutrition in it” (MWRA_FGD_01) and others identified specific micronutrients, “here on the packet, it is mentioned that there are a lot of vitamin elements in this product, such as Vitamin A, Vitamin B2, Vitamin B6 and a lot more nutritious content” (MWRA_TIPS_03). Women did not express any questions or concerns with how to use the product and shared that the pictorial instructions were helpful: “Anyone can easily understand by seeing the pictures how to eat this” (MWRA_TIPS_01).

HCPs inspected the packaging closely and considered the ingredients and images as appropriate for the target population. Though most of the labelling on the packet was written in both Bangla and English, the nutritional facts were solely in English and health care providers recommended these be provided in Bangla such that more women and their households would be able to read. When reading the micronutrients, one BRAC worker identified iron and zinc, and expressed concern that women may not like the product in the way that some pregnant women do not tolerate iron or zinc tablets: “As there is iron and zinc in it, then definitely it must taste a bit ‘*koshta koshta*’ (metallic taste)… And if it tastes ‘*koshta*’, meaning like iron or zinc tablets, then they have ‘*oruchi*’ (lack of appetite). Then they say, ‘*apa* (sister) I can't eat this, I feel bad if I take it, I feel nauseous’” (HCP_FGD_01). However, after tasting the product, the health worker considered the BEP as ‘*mishti mishti*’, or sweet‐like, adding, “their [women's] interest to eat this will increase” (HCP_FGD_01).

### 3.3 Household and community factors for adoption of BEP

Women and HCPs described considerations apart from the product itself that could influence the adoption and acceptance of BEP within households and the larger community. These included trust in the provider of BEP, the perceived health benefit of supplementation, cultural norms related to supplement use in pregnancy, and product sharing with family members.

#### 3.3.1 Provider trust

The JiVitA project has been conducting nutrition related research studies in the Gaibandha district for over 20 years, providing different formulations of nutritional supplements to women and infants. Throughout these studies, local female community members have been employed through JiVitA to collect data and provide supplements and counselling in participants' homes, making JiVitA a household name in the community. Women expressed trust in JiVitA as a nutritional supplement provider, since most had prior experience with the organisation, as exemplified by a woman in the TIPS group: “If you give it to them, they will eat it, they will say that ‘They (JiVitA) have given it for our (pregnant woman's) benefit, they will not want anything bad for us’” (MWRA_TIPS_02). Similarly, a woman mentioned that her husband encouraged her to take the supplement since it was given to her by JiVitA workers: “On the very first day, when he saw it, he told me that they (JiVitA) gave it for your good, you need to eat it as per their suggestions on time” (MWRA_TIPS_01), indicating the importance of provider trust in receiving buy‐in from family members.

#### 3.3.2 Perceived health benefits

When the BEP product was first introduced to focus groups, women and HCPs alike were concerned with understanding the benefits and potential risks of consumption. Women asked what would happen if they consumed BEP, how it would be helpful in pregnancy, and whether there were any harms. HCPs asked similar questions, and wanted to know what recommendations they should make in the event of illness, as exhibited by this quote: “If the mother consumes it [BEP] and gets sick, what would we do then? What advice would we give them? We need to know these things. We have to know both the advantages and the disadvantages” (HCP_FGD_01). In the follow‐up focus groups after the 2‐week trial period, none of the women reported any perceived ill effects and as one woman stated, “there was nothing to fear about the product” (MWRA_TIPS_02).

Despite having questions at the time of introduction, women and health providers shared the belief that if the BEP product was good for the pregnant woman's health, then it would benefit the growing child. As one woman put it, “Suppose those who will eat it [BEP], they will eat thinking that ‘I will stay healthy, so that my baby will stay healthy as well’” (MWRA_TIPS_02). One health worker attributed specific health benefits to the infant and mother with BEP consumption: “They will definitely eat it [BEP] because both mother and child will be healthy, the child will develop, the child will become more intelligent, the mother's physical strength (‘*shorirer goti*’) will be a little better than before, her immunity will increase” (HCP_FGD_01). The perception that BEP was a healthy product for the benefit of mother and child was echoed across the focus groups and participant types, contributing to its overall acceptability.

#### 3.3.3 Cultural norms

While BEP received a positive reaction among participants, health workers reflected on previous experiences introducing other nutritional supplements in their communities and warned that cultural beliefs or norms could influence the uptake of BEP. One health worker discussed the example of iron supplements: “Still there are many mothers who don't take iron because they say that the child will be too large; the child will not be born at home, you have to cut her (C‐section). This work [BEP distribution] will be like that; pregnant mothers will have worries and hesitations about what will happen if they eat this food” (HCP_FGD_01).

Women themselves did not express fears about consuming BEP specifically, but they acknowledged there could be resistance to adopting the product, particularly from mothers‐in‐law: “There may be obstacles. Many may say, it's good if we eat this, others will think, what will happen if we eat this? Many will comment a lot, in many homes people will say it is not good to eat this thing. The mothers‐in‐law will not understand what happens if pregnant women eat this” (MWRA_TIPS_02). As women in the study area generally live with their in‐laws, the mother‐in‐law's opinion may influence whether a daughter‐in‐law consumes BEP. In one focus group, women stressed that promotion of the product at the household level would be important in overcoming this challenge: “When you go door to door, the mothers‐in‐law will see you and then you can explain [what BEP is] to everyone, then they will understand that it is a really good thing” (MWRA_TIPS_02).

#### 3.3.4 Sharing and family member approval

**Figure 2:**
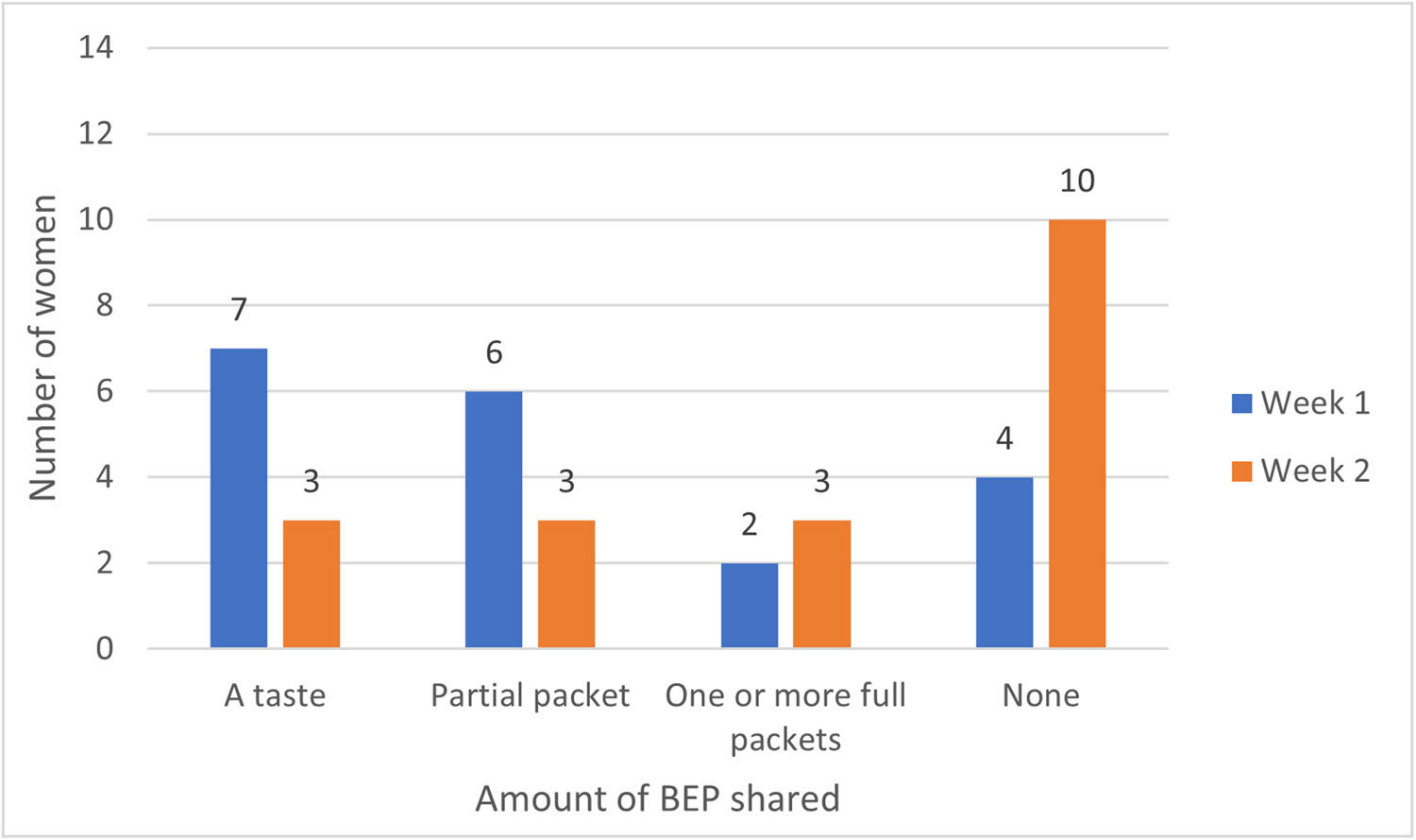
BEP sharing practices among TIPS participants, by week. Legend: The number of women who reported sharing some amount of BEP (a taste, partial packet, one or more full packets, none) in week 1 on the left‐side bar and week 2 on the right‐side bar. BEP, balanced energy protein supplement; TIPS, trials of improved practice.

Sharing, or giving some of one's food supplements to family members or friends for their personal consumption, may be a barrier to optimal adherence by the target recipient. Over the 2‐week period where participants were asked to consume BEP daily, 13 of 16 women reported some amount of sharing. However, most sharing occurred in the first week, and the amount shared was most often reported as “just a taste” (Figure 2). The recipients were most commonly mothers‐in‐law, children, or other adult family members.

Women discussed receiving approval for consuming BEP after family members tasted the product and/or read the label and concluded that the product would be beneficial and safe. This finding was reflected across all follow‐up focus groups, and highlighted by this woman's experience: “They (family members) ate and tasted and said, ‘this is a good thing, a ‘*pushtikor*’ (nutritious) thing, this can be eaten,’… My husband read and said, ‘here is written that this is nutritious food for pregnant women. If the baby is in the womb, both the mother and the baby will benefit’” (MWRA_TIPS_02).

Only two women reported giving away full portions of the product, which in each case were given to pregnant family members. Both women described how they offered the packets because they believed their pregnant relatives would be benefitted, as illustrated by one: “I gave it to my sister‐in‐law. Yes, as she is pregnant that's why I gave her a full packet. I gave it to her because she will be benefitted by that. I gave it to her willingly” (MWRA_TIPS_03).

When asked about how to reduce sharing with others, women identified counselling from the provider of BEP as the best strategy: “Those who provide this food to the pregnant mother should also explain to her that you can't share it with any others. If you eat it by yourself, then you will have nutrition and your child will also have the same. Only then they can understand that I have to eat the whole thing by myself without sharing it with anybody… Eat by yourself, and you will be benefitted” (MWRA_TIPS_02). For a few women, the original instructions given to them dissuaded them from sharing at all: “I didn't share it with anyone. I have a younger brother, he wanted it. I didn't give him. I said, ‘You can't eat this, madams said not to give it to anyone.' He didn't taste even a little bit” (MWRA_TIPS_03).

### 3.4 Utilisation and adherence strategies

**Figure 3:**
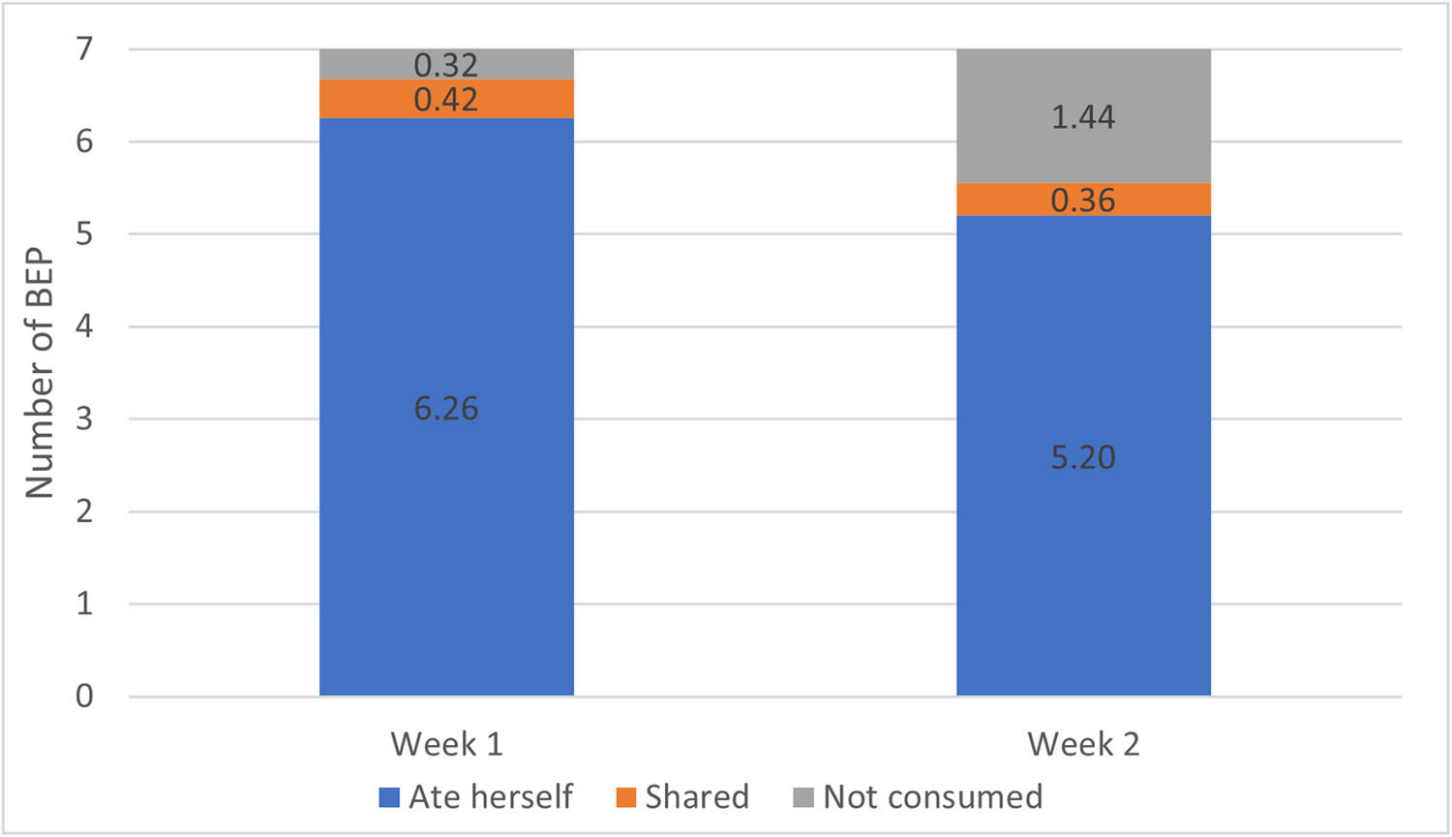
Average BEP utilisation among TIPS participants, by week. Legend: The average amount of BEP packets that were reported as eaten by the woman herself (blue), shared (orange), and not consumed (grey) in week 1 on the left‐side bar and week 2 on the right‐side bar. BEP, balanced energy protein supplement; TIPS, trials of improved practice.

Among women who participated in the TIPS activity the average total consumption over 2 weeks was 11.5 packets, or 82% of the 14 daily servings provided (Figure 3). In the follow‐up FGDs, women provided information on their experience consuming the supplement every day, including their perceptions on the portion size and timing. They also identified strategies that helped them, and could help future beneficiaries, with daily adherence.

#### 3.4.1 Portion size and timing of consumption

In general, participants stated that the portion size of the BEP supplement was appropriate as a daily serving, particularly if it could be consumed over the course of the entire day, as described by one participant: “If the food is given in this amount, then everyone will like it. If we want to eat it (at once), then we can. And if we think that we want to keep it for later, that is also possible” (MWRA_TIPS_03).

No participants perceived the daily portion as too small, though some women felt the daily portion could be reduced. To consume one packet per day, women reported consuming BEP at different times of day and over different durations. One TIPS participant described her experience, “As per the guidelines I tore those packets in the morning, and I took it little by little, all day long. I finished a packet in a day. On some days, I opened the packet at 8:00 am in the morning, on other days at 9:00 am in the morning; whenever I remembered. It took Asor or Maghrib prayer time for the packet to be finished” (MWRA_TIPS_02).

While most women appeared to like the flexibility regarding timing of consumption, concerns about eating the full amount in 1 day led some women to find it difficult to adhere: “Suppose I forgot to take this in the morning and remembered (in the afternoon); if I opened it at that time, I wouldn't be able to finish it. That's why I decided not to tear the packet open at that time” (MWRA_TIPS_02). This concern about wastage was not a common theme, but some women proposed packaging with a seal or cap so that a partially consumed packet could be hygienically sealed and saved for later. Whether such packaging could potentially de‐incentivize consumption of a full serving per day was not explored.

#### 3.4.2 Adherence strategies

Over the 2‐week consumption period, some women reported not consuming BEP on one or 2 days, due to forgetfulness, travel, work, or household chores. As one woman described, “Many people go to visit other places quite suddenly and they won't remember to take this with them as they are in a hurry. Or on some other days, they may be busy with work for which they forget to eat the food. All these things will cause delay” (MWRA_TIPS_02). However, the TIPS focus group participants identified various strategies that either helped them remember to eat BEP daily or could help future beneficiaries adhere, including: (1) keeping BEP packets within eyesight in their homes; (2) family members reminding them to consume them; and (3) receiving phone calls or text reminders from providers. Illustrative quotes of these commonly identified strategies are provided in Table 2.

**Table 2:** Adherence strategies identified by participants.

Adherence strategies	Illustrative quotes
1. Visual reminders	*“I kept that inside my room so I could see it in front of me on my way to and from the room. I kept it in eyesight.” (MWRA_FGD_TIPSFU_01)*
	*“There is a sewing machine located in‐front of my door, I kept the product in‐front of the machine. I took it out, and ate from there.” (MWRA_FGD_TIPSFU_01)*
	*“I always kept the packets on the table. After putting them into the jar I kept that on the table so that it will always be in my eyesight. So I always felt interested in eating. Like, the thing is in front of me that's why I need to eat.” (MWRA_FGD_TIPS_03)*
2. Family member reminders	*“I requested everyone in the house so that someone would remind me a little. Every morning, someone or the other reminded me and I took it then. Or I remembered myself. I told everyone; my nonod (husband's sister), my Ja (husband's brother's wife), my shashuri (mother in law). I told them to remind me in the morning.” (MWRA_FGD_TIPS_02)*
	*“The children can also do that. If the mother forgets, the child can say, ‘Ammu here is your food. You need to eat that.’ The children can also help give reminders. (What aged children can help give reminders?) Children of 10‐12 years old can help give the reminders.” (MWRA_FGD_TIPS_01)*
	*“My husband reminded me after I brought the food. He says ‘eat it’, or ‘you have to eat it, or ‘did you have it or not’. Yes, He reminded me every day. Yes, my mother‐in‐law can remind me.” (MWRA_FGD_TIPSFU_02)*
	*“Sometimes someone reminded me to eat on time. Then I ate. They said ‘the packet was provided to you, it should be eaten on time’. And then I could remember and ate then. My husband and my mother‐in‐law reminded me. Those who were aware about this food, reminded me almost all the time.” (MWRA_FGD_TIPS_03)*
3. Phone call or text reminders from providers	*“If you give it that way (if you send reminder messages on the phone) it will also be good. The message will be sent to your phone, then you will remember, oh, I need to eat it. If you message like that, she will remember and she will instantly eat it. Or when the message comes that time she can start to eat, and continue to eat that all day long. Sending messages every morning will be good for everyone. Because sometimes even the mother‐in‐law or sister in law may forget; then, if you see the message, you will remember. That will be good.” (MWRA_FGD_TIPSFU_02)*
	*“You can call and say ‘‐‐‐‐‐‐ apa, did you eat? If not then please eat the food on time’. That's all. Those who are literate, can read this Bangla (SMS). (For a woman who can't read) She can come to know by someone in the house who can read. If you call once a week then it will be remembered. And while you are giving a phone call that time she will think about the importance of the food. Yes you can do like that (as JiVitA workers did in those two weeks). When they reminded me, I definitely ate those foods.” (MWRA_FGD_TIPSFU_03)*

## 4 DISCUSSION

In this analysis, we explored the acceptability of a new BEP product among Bangladeshi WRA and their HCPs as well as the feasibility of adhering to daily supplementation, with the aim of informing intervention delivery and communication in a subsequent trial.

Overall, the BEP supplement was well accepted by the study participants. Contributors to acceptability included the product's taste, informative labelling and packaging, perceived nutritional value and health benefit, trust in the provider, and family member approval. Potential barriers to acceptability were related to cultural norms around supplement use during pregnancy, and mixed perceptions on the texture and smell of the product. Women felt the portion size was acceptable and could be consumed daily throughout pregnancy, and they identified several strategies to optimise adherence.

Hedonic testing of different food products for pregnant women, including BEP, lipid‐based nutrient supplements (LNS), and RUTF has been conducted in various settings, including Nepal (Lama et al., 2022), Bangladesh (Ali et al., 2015), Niger (Isanaka et al., 2019), Malawi and Ghana (Klevor et al., 2016). Findings from these studies indicate that these products are generally well accepted, with sensory attributes being a critical component of acceptability and subsequent adherence. In our study, the taste of BEP was well liked and the ingredients were recognisable, which increased women's confidence in the product; however the lentil smell and thick texture were identified as potential barriers to use. The research team has since worked with the manufacturer to develop a product with a milder smell and a slightly thinner consistency for the future trial. These findings underscore the importance of piloting BEP products in the target population before administering them, as this enables the researchers to return to the manufacturer to develop a more acceptable product.

The 2‐week TIPS activity demonstrated that sharing of BEP with family members was common, likely due to the curiosity of the household members to try the product. However, the majority of sharing occurred in the first week, when the product was a novelty item, with significantly less sharing in the second week. Moreover, the actual amount of BEP that was shared was minimal, as most women reported family members having “just a taste” and seldom more than half a packet. Minimal to moderate amounts of sharing with family members was observed in other BEP acceptability studies (de Kok et al., 2021; Eglovitch et al., 2021). A pilot study conducted in Nepal, in which pregnant women were provided BEP for 8 weeks, similarly found that sharing was most common in the first week of the programme, and that the total amount of sharing was limited thereafter (Lama et al., 2022). In Burkina Faso, sharing intentions were recorded as low after pregnant women received clear instructions on why women should not share their BEP portions with others (de Kok et al., 2021). While some sharing, or tasting, is to be expected, clear identification of the beneficiary, combined with communication messages on the benefits of consumption in pregnancy are likely to reduce sharing and improve uptake. In the trial, we plan to provide additional packets to women at their first supplementation visit to allow for family members' tasting and approval while ensuring the women themselves have enough quantity of product for daily use.

Interestingly, the only instances of full packet sharing, or giving away packets, were reported by two participants who visited pregnant family members and identified these relatives as beneficiaries. This suggests that women's acceptance of BEP was in part related to the perceived health benefits for the pregnant woman and the unborn child. This theme was reiterated in participants' understanding and appreciation of the product labelling. The image of the pregnant woman on the packet and emphasis on the micronutrient content reinforced the intended target group. Effective marketing of the product, including designation of the beneficiary and nutritional content, will be helpful strategies for future BEP supplementation programmes. Promotion and understanding of the positive health benefits has been identified as a key element of acceptability of maternal nutritional supplements in many settings (Harding et al., 2014; Klevor et al., 2016; Lama et al., 2022; Young et al., 2010). In Nepal, the identification of BEP as ‘both food and medicine’ dissuaded sharing with children and underscored the perception of BEP as a beneficial food product for pregnant women only (Lama et al., 2022).

Our findings suggest that in this context, communication related to BEP should also address fears about side effects and myths regarding large babies and cesarean deliveries. While women themselves did not report such concerns in our study, HCPs emphasised these perceptions as a potential barrier, and similar misconceptions about nutritional supplements and food intake have been documented in South Asia (Ara, 2013; Hutter, 1996; Nisha et al., 2019) and other regions of the world (Clermont et al., 2018; Maggiulli et al., 2022; Zerfu et al., 2016). However, studies have indicated that reduced food intake in pregnancy is more closely linked to women experiencing illness, food aversion, or loss of appetite rather than an intentional behaviour to reduce the size of the newborn (Christian et al., 2006; Harding et al., 2017). Addressing these common ailments during pregnancy, through antenatal care services and counselling, would likely help pregnant women manage these symptoms and improve supplement adherence. Additionally, community or household‐level counselling and promotion of BEP could help providers answer common questions about supplementation as well as reduce fears and combat myths.

Trust in the provider was another important component of acceptability and adoption. Trust in JiVitA as a provider of nutritional supplements led participants and their family members to readily accept the product. Another study in Bangladesh similarly found that community health worker credibility was a facilitator in garnering support from husbands and mothers‐in‐law for nutrition‐related behaviour change, and conversely, that a lack of credibility could undermine the CHW health messages (Wable Grandner et al., 2022). For the trial, local JiVitA CHRWs will distribute the product door‐to‐door along with a standard counselling module on nutrition in pregnancy. The participants consuming BEP will receive additional counselling on the benefits of BEP and instructions for use. An important step needed to translate trial findings into an eventual BEP programme will be considering the role of different trusted providers of health information to communities, such as antenatal care providers or pharmacists, and exploring how these stakeholders can be engaged in promoting or distributing BEP to beneficiaries and how they can be equipped to answer questions about the product.

Reflecting on their experiences from the TIPS exercise, participants identified several strategies that helped them remember to consume the product daily, including placement of BEP in a location that they would remember, having family members remind them to consume it, and receiving calls or text messages from BEP providers. Use of adherence partners has been shown to be an effective and acceptable strategy to support adherence to micronutrient supplementation in Ethiopia and Kenya (Martin et al., 2017) and this strategy could be translated to BEP supplementation. mHealth interventions, including text messages to women, have shown promise in improving adherence to nutritional supplements (Soepnel et al., 2022). In the trial, we plan to leverage an existing tool, the JiVitA calendar, that was previously used by participants for tracking their menstrual periods. Participants will receive a physical calendar and instructions on how to mark the days when they consumed BEP. In this way, the calendar will serve as both a visual reminder for daily consumption and a tool to facilitate recall. Such strategies will need to be evaluated within the context of BEP, and the potential limitations of the approach need to be assessed in different settings.

The primary limitations of this study are the relatively small sample size and the short duration of the TIPS activity. Although high adherence rates were promising, the small sample size makes it difficult to evaluate whether the findings around utilisation and adherence are generalisable in the longer term. It is possible that consuming the product over a longer period could become mundane to women. However, other formative studies that conducted BEP acceptability testing over 8–10 weeks reported high levels of compliance throughout (de Kok et al., 2021; Lama et al., 2022). Additionally, our participants included WRA, and not solely pregnant women, making these results non‐specific despite the intervention being targeted for pregnant women. However, we purposely sampled lactating mothers who had recently given birth as they could call on their recent experiences when answering questions.

The mixed methods design used to assess the acceptability and feasibility of BEP supplementation among WRA and their health providers is a key strength of this study. Providing health workers with the opportunity to taste and evaluate the BEP beyond the product label contributed to their buy‐in and may be leveraged in future programming. FGDs highlighted the explicit factors that contributed to the acceptability and adoption of the product, expanding beyond the sensory attributes of the product, and including household and community level factors. The TIPS activity demonstrated the feasibility of the intervention, showing that high adherence is possible, at least in the short term, and informed communication strategies to be adopted in the subsequent trial. Additionally, by conducting FGDs before and after the TIPS, we were able to observe how initial perceptions towards the product shifted positively with familiarity and continued use.

The findings of this formative study have meaningful implications for development of the effectiveness trial and for future BEP programmes. In the trial, we plan to use the well‐accepted BEP supplement, slightly modified in smell and texture, and with two flavours to provide women with more than one option. The labelling and packaging will remain the same, and counselling provided to women receiving the supplement will include a description of the benefits, clear identification of the target beneficiary, instructions for use, and adherence strategies discussed previously. The trial will shed light on long‐term adherence to such supplements and their effect on birth outcomes through different targeting strategies. Outside of the trial, findings of this study contribute to existing literature and may help to inform future programming for BEP supplementation in Bangladesh, especially with regards to components of product acceptability and communication strategies.

## 5 CONCLUSION

We found the BEP product to be well accepted, in terms of both taste and its perceived health benefits which were conveyed through product labelling and packaging. Despite some concerns about the product smell and texture, this did not appear to deter women from consuming the supplement, as evidenced by the high adherence in the TIPS group. Other components of acceptability included the importance of cultural beliefs related to using supplements in pregnancy, family members' tasting and approval of the product, and trust in the provider. Additionally, the women identified several important adherence strategies that will inform counselling and recommendations in a subsequent effectiveness trial. This study demonstrates how mixed methods research can yield valuable insights into the acceptability and adoption of nutritional supplements in future studies and programmes.

## AUTHOR CONTRIBUTIONS

**Parul Christian**, **Andrew L. Thorne‐Lyman**, **Anna Kalbarczyk**, and **Eleonor Zavala**: designed the study and the protocol. **Nazrana Khaled** and **Atiya Rahman**: trained the field data collectors. **Hafizur Rahman**, **Hasmot Ali**, **Kaniz Ayesha**, **Rezwanul Haque** and **Towfida J. Siddiqua**: coordinated the implementation of the study and field data collection. **Eleonor Zavala**, **Atiya Rahman**, **Nazrana Khaled**, **Barnali Chakraborty**, **Anna Kalbarczyk**, and **Andrew L. Thorne‐Lyman**: analyzed and interpreted the data. **Eleonor Zavala**: drafted the manuscript, with significant support from Atiya Rahman. All authors, including Kaosar Afsana and Mary de Boer provided critical feedback and approved the final version.

## CONFLICT OF INTEREST STATEMENT

The authors declare no conflict of interest.

## Supporting information

Supporting information.

## Data Availability

The data that support the findings of this study are available from the authors upon reasonable request.
