# Supplementary material for: Acceptability of a balanced energy protein (BEP) supplement for pregnant women in Bangladesh
Source: Matern Child Nutr. 2023 Nov 22;20(Suppl 6):e13587. doi: 10.1111/mcn.13587 (PMC11439734; doi:10.1111/mcn.13587)
Supplement: Supplementary file 1 — Supporting information. [file MCN-20-e13587-s001.docx]

Supporting Information: Acceptability of a Balanced Energy Protein (BEP) supplement for pregnant women in Bangladesh

**Table of Contents:**

Semi-structured facilitator guide for FGD: Married women of reproductive age….….2

Semi-structured facilitator guide for FGD: Health workers…………………….……….…..…6

Semi-structured facilitator guide for TIPS FGD…………….…………………….….……………..10

Phone Adherence and Sharing Assessment…………….…………………………….……..……..12

Code List………………………………………………………………………………………………..……………14

COREQ Checklist………………………………………………………………………………………….………16

**JOHNS HOPKINS BLOOMBERG SCHOOL OF PUBLIC HEALTH**

**SEMI-STRUCTURED FACILITATOR GUIDE FOR FOCUS GROUP DISCUSSION:**

**Married women of reproductive age**

**Study Title:** Balanced energy protein (BEP) implementation research in rural Bangladesh

**Principal Investigator:** Christian

**IRB No.:**

**PI Version Date:** Sept 1, 2021 – Version 1

**Week of FGD: Date:**

**yy**

**mm**

**dd**

**Facilitator ID: Initial:**

**Notetaker ID: Initial:**

**Location: Number of participants:**

**Focus group start time: Focus group end time:**

**hh mm hh mm**

**Demographic Information**

1. This information will be collected individually after the consent process.

| Woman ID | 1a. Name of Participant | 1b.  Age  (years)  18-35= years  99= Don’t know | 1c. Number of children  0-7= no of children  8=8 or more  9=don’t know | 1d. Currently pregnant or lactating (6 months)?  0=No  1= Currently pregnant  2=lactating  9=Don’t know | 1e. Literacy  (Can you read or write a letter in Bangla?)  0=No  1=Yes  9=Don’t know | 1f. Education  (completed highest class of school)  00=No schooling / not applicable  01-09= Number of class  10= SSC/Dakhil passed  11= 11^th^ class completed  12= HSC/Alim passed  13= Hon’s/Degree 1^st^ year completed  14= Hon’s/Degree 2^nd^ year completed  15= Degree-pass/Fazil/ Hon’s 3^rd^ year completed  16= Hon’s/ Previous  17= Master Degree/ Kamil or Higher  18= Diploma (vocational/paramedic etc.)  19= Professional Degree (*MBBS / ENGR / LLB* etc.)  77=Non-formal education  99=Don't know | 1g. Occupation  0=No occupation  1=Work on own farm / as share cropper  2=Day/ unskilled laborer (agricultural & migrant etc)  3=Maid servant / Fisherman  4=Contracted/skilled laborer (long term domestic or agricultural)  5=Own business  6=Private service (salaried, skilled factory and office workers. salesperson, skilled laborer etc)  7=Government service (all GOB-paid employees)  8=Other  9=Don't know |
| --- | --- | --- | --- | --- | --- | --- | --- |
|  |  |  |  |  |  |  |  |
|  |  |  |  |  |  |  |  |
|  |  |  |  |  |  |  |  |
|  |  |  |  |  |  |  |  |
|  |  |  |  |  |  |  |  |
|  |  |  |  |  |  |  |  |
|  |  |  |  |  |  |  |  |
|  |  |  |  |  |  |  |  |
|  |  |  |  |  |  |  |  |
|  |  |  |  |  |  |  |  |

**Introduction**

*Welcome. My name is __________ and I will be facilitating our discussion today.* *The purpose of this group meeting is to discuss special food snacks for pregnant women in Bangladesh. We are interested in your opinions and your ideas on this topic, so you should feel comfortable talking freely. When I ask questions, I am addressing all of you and I encourage you to respond to other members’ comments. You could agree, disagree, or share a different experience. We want to ensure we are getting a variety of points of view, so it is ok to provide a different answer – there is no right answer to any question. Before we start, let us introduce ourselves with our name. If everyone is ready let’s begin.*

**Diet and food products during pregnancy**

1. Let’s first start with talking about diet and food intake when a woman is pregnant. *S*ome say women need to “eat for two” during pregnancy. How do you feel about this?

*Additional questions:*

- *What are some ways in which pregnant women increase their food consumption?*
- *What are the challenges to eating more during pregnancy? [Probe: Any others?]*
- *What are special snacks pregnant women should eat? How are they special?*
- *Have you consumed any special snacks or foods while pregnant? Please describe them.*
- *What do you think of packaged foods that are meant to be eaten by pregnant women? Do you know of any such foods?*
- *What would be some advantages of such snacks? Disadvantages? [Probe: What else?]*

1. Here are some real samples of a special food product that has been developed for pregnant women. It contains rice and dal (lentil) and vitamins. Each packet is like a snack to be eaten daily during pregnancy and in addition to your daily meals. We would like you to look at it and taste and let us know what you think about it. [*pass out a sample of one sachet per person*].

- *What do you think about the look of the packet? Can you describe what you see? What do you think about the label? What does it tell you?*
- *What do you think about the taste of the product? And its smell and consistency? How would you change it? What do you like? What do you not like?*
- *Do you think you would be able to eat one packet each day during your pregnancy? What kind of challenges or concerns would you have about that?*
- *Is this the right amount for a daily snack between meals? Is it too much, too little? Why?*
- *What kind of questions about this product do you think women would have if it were being given out as a program?*

**Delivery methods**

1. We would like to find out the best way to give this food product to pregnant women. We have been thinking about different ways to deliver these to women when they are pregnant and want your opinion about what will work or not.

[For each ask w*hat are the advantages of distributing using the option being discussed? Are there any disadvantages? What else?]*

- 1. *First let’s discuss the possibility of receiving these foods during an antenatal checkup. What do you think about that idea?*
  2. *Second, what about if a health worker delivers it when she comes to visit at home? Let’s discuss this.*
  3. *Third, what if these food packets were provided through a pharmacy nearby your home? This would be done by providing you a voucher. Let’s discuss this.*

1. Now we want to know what amount would be good to deliver or provide at a time.

Do you think giving (a box of) 30 of these packets to women each month would work? How about 60 packets every 2 months? What other amounts do you think would be better?

- *Are there any challenges that you might face in going to pick up the supply of packets each month if you went to health checkup or the pharmacy by yourself? What are they? Are they different for the smaller box vs. the bigger box?*
- *Are there other people in your family who could help pick them up? Who? Are there any challenges that they might face?*
- *What do you think are ways by which you may run out of supply? How could those be addressed?*

**Targeting foods to undernourished women**

1. Are there some women in the community who you think might benefit more than others from a food like this? Who are they likely to be?
2. Some programs may use a woman’s weight to identify those with low weight who should receive such products.

- What do you think about that idea?
  - What do you think about the idea of weighing women during pregnancy to help decide who to give it to? What might be the advantages of doing this? Are there any disadvantages? [Probes: why? how?]
  - Do you think there could be any problems to give it to some women but not others? What would those be?

7) Is there anything else you would like to share with me today?

Closing script: Thank you all for your time and sharing your experiences with me today.

**Introduction of food packets [For the two FGDs in which women are participating in the “TIPS”]**

*We are now giving you enough of these packets for two weeks. We would like you to consume one each day. It may help you to consume it at the same time of day, for example after dinner or in the afternoon. You can also ask someone in your family to remind you to eat it. This food is for you not for other people in your family so please do not share it with others. We also have this pamphlet explaining more about the food product and how it should be consumed and* **JOHNS**

**HOPKINS BLOOMBERG SCHOOL OF PUBLIC HEALTH**

**SEMI-STRUCTURED FACILITATOR GUIDE FOR FOCUS GROUP DISCUSSION:**

**Health workers**

**Study Title:** Balanced energy protein (BEP) implementation research in rural Bangladesh

**Principal Investigator:** Christian

**IRB No.:**

**PI Version Date:** Sept 1, 2021 – Version 1

**Week of FGD: Date:**

**yy**

**mm**

**dd**

**Facilitator ID: Initial:**

**Notetaker ID: Initial:**

**Location: Number of participants:**

**Focus group start time: Focus group end time:**

**hh mm hh mm**

**Demographic Information**

1. This information will be collected individually after the consent process.

| 1a. Name | 1b.  Age  (years)  18-98= years  99= Don’t know | 1c. Education  (completed highest class of school)  00=No schooling / not applicable  01-09= Number of class  10= SSC/Dakhil passed  11= 11^th^ class completed  12= HSC/Alim passed  13= Hon’s/Degree 1^st^ year completed  14= Hon’s/Degree 2^nd^ year completed  15= Degree-pass/Fazil/ Hon’s 3^rd^ year completed  16= Hon’s/ Previous  17= Master Degree/ Kamil or Higher  18= Diploma (vocational/paramedic etc.)  19= Professional Degree (*MBBS / ENGR / LLB* etc.)  77=Non-formal education  99=Don't know | 1d. Type of provider  00=Untrained TBA  01=Village doctor/ Kabiraj/ Shaman  02=Trained TBA  03=Ayurvedic doctor/ Homeopath  04=BRAC CHW  05=Smiling Sun CHW  06=Marie Stopes CHW  07=Any other NGO clinic provider  08=Paramedic/ SACMO/ MA  09=FWA/FWV  10=Nurse/Midwife/SBA  11=MBBS Doctor  99=Don't know | 1e. Facility/place of work  00=At woman’s home  01=FWA/FWV’s home  02=NGO clinic  03=Private chamber  04=Private clinic or hospital  05=Government community clinic/ FWC/ Satellite clinic/ sub-center  06=Thana Health Complex  07=MCWC  08=Government District Hospital  88=Other  99=Don't know | 1f. Number of years of work  1= 1 year or less  2-30= years  99= Don’t know |
| --- | --- | --- | --- | --- | --- |
|  |  |  |  |  |  |
|  |  |  |  |  |  |
|  |  |  |  |  |  |
|  |  |  |  |  |  |
|  |  |  |  |  |  |
|  |  |  |  |  |  |
|  |  |  |  |  |  |
|  |  |  |  |  |  |

**Introduction**

*Welcome. My name is __________ and I will be facilitating our discussion today.* *The purpose of this group meeting is to discuss special food snacks for pregnant women in Bangladesh. As health care workers, we are interested in your opinions and your ideas on this topic, so you should feel comfortable talking freely. When I ask questions, I am addressing all of you and I encourage you to respond to other members’ comments. You could agree, disagree, or share a different experience. We want to ensure we are getting a variety of points of view, so it is ok to provide a different answer – there is no right answer to any question. Before we start, let us introduce ourselves with our name. If everyone is ready let’s begin. Before we get started, I would like everyone to introduce themselves with their name and where you work.*

**Antenatal care and role as provider**

- I want to learn about all your experiences providing health care to pregnant women.
- *Would you share your experience providing health care to pregnant women like? What do you enjoy? Why? What are some of the challenges? [Probe: Time, support, training]*
- *Can you describe what sorts of things you do when a pregnant woman comes in for a health checkup?*
- *Do you take women’s weight at health visits? Why or why not?*
- *Do you take women’s height at health visits? Why or why not?*

**Special foods for pregnant women**

- It is important that women have a healthy and nutritious diet during pregnancy. I would like your opinion about this and special food products for pregnant women.
- *What are some ways in which pregnant women in the community increase their quantity and quality of food consumption?*
- *What are the challenges to eating more and nutritious foods during pregnancy? [Probe: Any others?] How do you think women overcome the challenges that they face related to this?*
- *What are special snacks pregnant women should eat? How are they special?*
- *What do you think of special foods that are meant to be eaten by pregnant women? Do you know of any such foods that are commonly prepared at home? What would be some advantages of such snacks? Disadvantages? [Probe: What else?]*

3) I will now introduce you to a new product for pregnant women. Research has shown that eating these kinds of snacks in pregnancy helps mothers grow a healthy baby and reduces the risk poor birth outcomes. Previous nutrition programs in Bangladesh have used similar products before, such as Pushti packets. Please take a moment to look at the product and taste if you want.

- *What do you think about the product? What do you like or not like? How would you change it?*
- *Do you think pregnant women will want to eat this? Why or why not?*
- *Would you feel comfortable recommending this product to pregnant women? Why or why not?*
- *Is this the right amount for a daily snack between meals? Is it too much, too little? Why?*
- *Are the instructions on the package clear? How would you change it? What other information would be important to know?*

**Delivery of food snacks**

1. We would like to find out the best way to give food snacks to pregnant women. What are some ways these food products could be provided to pregnant women? (Allow an open-ended discussion. Probe with different options)

*For ANC/CHW women’s group distribution:*

- *Would you expect women to come to the clinic regularly for the products? Would they need to be delivered to their home? How often?*
- *What are the advantages/disadvantages of each approach?*
- *In your opinion, wow feasible is it for health workers to provide this intervention?*
- *Is there enough storage for these products at your place of work?*
- *What concerns or questions do you have about providing the intervention to pregnant women? [Probes: Why is this a concern? Could you give examples]*

*For pharmacy or market vendor distribution:*

- *What are the advantages/disadvantages of a pharmacy pick-up or market pick-up approach? Especially compared with ANC visits or other health workers?*
- *How successful do you think would a voucher system be? Are there other products sold using a voucher in your experience such as contraceptive methods?*
- *Do you think families would be willing to purchase these types of products if you had them in the pharmacy?*
- *What are the concerns if pregnant women had to travel to get the food packets?*

*Delivery channels to explore specifically if not brought up.*

- *During an ANC visit, given a 2-month supply.*
- *Via a CHW visiting them 1 or 2 times a month.*
- *Picking up at a pharmacy when convenient for the women, with redeemable vouchers.*
- *Picking up at a local market, with redeemable vouchers.*

**Targeting Approaches**

1. Who are the pregnant women you think would benefit the most from such foods? For example, there are some programs in your community that target women in poverty, such as the VGF women. [Probes: why? how do they benefit?)
2. Some programs may use a woman’s weight and height to identify those who should receive such products. What do you think about that idea?
   - *What are the advantages of that? What are the disadvantages? [Probes: why? how?]*
   - *Do you think that’s fair or unfair? Would it create problems if you were giving this to some but not other women in the same clinic? [Probes: why/how?]*
   - *Would you be willing to take weight and height during pregnancy to identify women most in need as part of your job? What would be some issues you would face? What kind of support would you need in doing this?*
   - *What instruments are available to you to do weight and height measurements? How feasible is it to do weight measures throughout pregnancy? What are the challenges? [Probe: working scale, why/how would you address the challenges?]*

7) Is there anything else you would like to share with me today?

Closing script: Thank you all for your time and sharing your experiences with me today.

**JOHNS HOPKINS BLOOMBERG SCHOOL OF PUBLIC HEALTH**

**SEMI-STRUCTURED FACILITATOR GUIDE FOR SECOND FOCUS GROUP DISCUSSION:**

**Married women of reproductive age selected for TIPS**

**Study Title:** Balanced energy protein (BEP) implementation research in rural Bangladesh

**Principal Investigator:** Christian

**IRB No.:**

**PI Version Date:** Sep 1, 2021 – Version 1

**Week of FGD: Date:**

**yy**

**mm**

**dd**

**Facilitator ID: Initial:**

**Notetaker ID: Initial:**

**Location: Number of participants:**

**Focus group start time: Focus group end time:**

| Names of participants in FGD |
| --- |
|  |
|  |
|  |
|  |
|  |
|  |
|  |
|  |
|  |
|  |

**Experience with use of BEP supplements:**

1. We are now going to discuss your experience of eating the food packets we provided you. Each of you took home the food packets for a couple of weeks to try. We would like to hear more about what you thought of the food packets, could you tell us about your experiences?

- *Could you tell us about any concerns that you had? What else? When did you generally eat this food?*
- *Did any of you not eat all the food that was given? How often did this happen? Why?*
- *Did any of you eat all of the food that was given, every day? How did you remember to eat it? Could you tell us about any difficulty you had remembering to eat the food every day?*
- *Were you able to finish the entire packet in a day? Was it hard to eat it all at once? Why? If not, how did you usually eat it during the day?*
- *Did you have any problems storing the food packets? If a packet was not consumed in one sitting, did you eat it later or did you discard it? Did you give it to someone else? How did you store an open packet?*
- *Do you have any ideas about ways that we could get women to better remember to eat the food every day?*
- *Did any you or any family members have any questions that came up about the foods? What were they?*
- *Did any of you share with family members? Why or why not? Who did you share it with? How many packets were consumed by others?*
- *Do you had any problems using the Pamphlet? Do you have any ideas of including any in this pamphlet for better understanding by consumer?*

1. If you were given this snack every day for 6 months, and be expected to eat it every day, how would you feel about that? Let’s discuss your thoughts and suggestions.

- *What would be the main concerns about eating this food every day for 6 months? What would be some positive things?*
- *If people say, no they wouldn’t eat daily - what could be the reasons for why they would not be able to eat this daily?*
- *What would make it easier to eat this regularly?*
- *Some women may forget to take this food. Who could remind you to eat it regularly? What do you think about text reminders?*
- *Some of you mentioned sharing the product with your children or other family members? We want this to be consumed only by pregnant women. What are some ways in which sharing could be reduced? Any others?*

**JOHNS HOPKINS BLOOMBERG SCHOOL OF PUBLIC HEALTH**

**PHONE ADHERENCE AND SHARING ASSESSMENT**

**Married women of reproductive age**

**Study Title:** Balanced energy protein (BEP) implementation research in rural Bangladesh

**Principal Investigator:** Christian

**IRB No.:**

**PI Version Date:** Sept 1, 2021 – Version 1

**Week of Assessment: Date:**

**yy**

**mm**

**dd**

**Interviewer ID: Initial:**

**Union**

**Sector**

**TL PIN**

**Mauza**

**HH ID: Woman UID:**

**Husband’s name**

**Woman’s name**

**Interview script:**

Hello, I am calling from the JiVitA project. The other day we gave you some food packets to try out and I am calling to find out about how you are consuming them.

1. *Can you tell me how many packets you have eaten/completed*

00-14 = number of packets

99 = don’t know

*in the past 7-days?*

1. *Can you count and tell me how many unopened packets do you have?*

1. *When was the last date you ate a packet?*

**dd mm**

1. *We would like to understand who else has eaten or tried the food. It is ok if others besides you have eaten it, we just would like to know more about how it has been shared. Please tell me about who else has tried/had the food packet besides you in the past week. [Probe: anyone else?] Record all responses.*

|  | **Did _____ try/have the product?**  **0= No, 1=Yes** | **How much did they try/have?**  **00.0=none**  **01.0-14.0=number of packets (including half packets, code as .5)**  **88.8=just a taste**  **99.9=don’t know** |
| --- | --- | --- |
| **Husband** |  |  |
| **Mother-in-law** |  |  |
| **Children** |  |  |
| **Other adult in the family** |  |  |
| **Neighbor/Friend** |  |  |

*answering some questions about it. After two weeks we will have the same group meeting again to discuss your experience with using this food product.*

*Do you have any questions for me? We thank you for participating in this activity.*

**Code List**

| Id | Parent Id | Depth | Title | Description |
| --- | --- | --- | --- | --- |
| 1 |  | 0 | Communities | Needs, resources, capacities, and other factors at the community level |
| 2 | 1 | 1 | Community Support | supports providede by neighbours, communities |
| 3 | 1 | 1 | Social and Cultural beliefs and norms | about food, pregnancy, etc. |
| 4 |  | 0 | Enabling environment | Policy frameworks, governance, finances and stakeholder dynamics that can affect ANC and BEP |
| 5 |  | 0 | Households | Needs, resources, capacities, and other factors at the household level. This could include decision-making, access to household finances, roles of husbands/mothers in law, etc. |
| 6 | 5 | 1 | Access to resources | This includes financial resources; food-sharing; access to transportation; etc. |
| 7 | 5 | 1 | Family Support | Supports provided/needed from family members |
| 8 | 5 | 1 | Norms (and practice?) | including household chores/responsibilities and inter-household relationships. |
| 9 | 5 | 1 | Power and decision-making |  |
| 10 | 9 | 2 | Agency | their ability to define and act on goals, make decisions that matter to them, and participate in the economy and public life |
| 11 | 9 | 2 | Mobility |  |
| 12 |  | 0 | Implementation outcomes | specific to BEP and ANC |
| 13 | 12 | 1 | Acceptability | of BEP and ANC and/or other delivery mechanisms. Someone discussing how they like something. |
| 14 | 12 | 1 | Adoption | of BEP and ANC and/or other delivery mechanisms |
| 15 | 12 | 1 | Appropriateness | of BEP and ANC and/or other delivery mechanisms. Perceptions around something being appropriate for a group of people. Can this intervention/delivery modality work within this context? |
| 16 | 12 | 1 | Costs | of BEP and ANC and/or other delivery mechanisms. May be financial or opportunity. |
| 17 | 12 | 1 | Feasibility | of BEP and ANC and/or other delivery mechanisms |
| 18 | 12 | 1 | Fidelity | of BEP and ANC and/or other delivery mechanisms. How closely to providers or women think they can follow BEP implementation procedures. Could include non-daily consumption, sharing, anything diverting from recommendations for use. |
| 19 | 12 | 1 | Penetration | of BEP and ANC and/or other delivery mechanisms. How much of the community can be reached? How many providers can be reached? etc. |
| 20 | 12 | 1 | Sustainability | of BEP and ANC and/or other delivery mechanisms |
| 21 |  | 0 | Implementation Processes | Initiation, planning, implementation, and sustaining interventions. Could include ANC, BEP, etc. These are process related factors, not factors inherent to the intervention itself. |
| 22 |  | 0 | Implementing organization | Ministries, NGOs, private sector - any comment about an organization itself. Perceptions of the organization and how it works. |
| 23 | 22 | 1 | organizational systems and structures | Are organizations structured such that they can conduct these interventions |
| 24 | 22 | 1 | Other key stakeholders for implementation | i.e. the role of the MOH, UNICEF, WHO, or other major players. |
| 25 | 22 | 1 | Staff considerations | i.e. personalities; bed-side manner; capacities; etc. |
| 26 |  | 0 | Individuals | Needs, resources, capacities and other individual-level factors |
| 27 | 26 | 1 | Individual attitudes | about BEP, ANC, pregnancy, weight gain, etc. |
| 28 | 26 | 1 | Individual knowledge/perception | About BEP, ANC, Pregnancy, etc. |
| 29 | 26 | 1 | Individual practices | about BEP, ANC, pregnancy, weight gain, etc. |
| 30 |  | 0 | Objects of implementation | Components of the intervention - i.e. BEP, its delivery mechanism, or targeting |
| 31 | 30 | 1 | ANC | as an intervention |
| 32 | 30 | 1 | BEP | anything about BEP itself |
| 33 | 32 | 2 | Adherence |  |
| 34 | 32 | 2 | Amount / Supply | Perceptions about the amount of product delivered/obtained at one time. How do women feel about a 30- or 60- or 180-day supply? How do providers or SMC workers feel about amount of supply at a given time? |
| 35 | 32 | 2 | Labeling / Packaging | Any descriptions or perceptions of the packaging and labeling of the product, whether positive, negative, or a suggested modification. |
| 36 | 32 | 2 | Portion size / Frequency | Perceptions about the portion size and/or frequency of consumption (every day). Could include perceptions about whether the portion size is too bif or small, whether the frequency is too much, too little, or could capture how women may choose to eat this product daily, ie eating half in the morning and half in the afternoon. |
| 37 | 32 | 2 | Sensations or discomforts | Any descriptions or perceptions about the physical sensations or discomforts experienced after eating or tasting the product. For example, whether they had a stomach ache or discomfort after eating, etc. |
| 38 | 32 | 2 | Sharing | Any descriptions or perceptions related to sharing of BEP. |
| 39 | 32 | 2 | Storage | any information/concerns related to how BEP should be stored |
| 40 | 32 | 2 | Taste / Smell | Any descriptions about the taste or smell of the product, such as what food it reminds them of. |
| 41 | 32 | 2 | Texture | Any descriptions about the texture of the product, such as it sticking to the roof of the mouth, whether it feels smooth or grainy, etc. |
| 42 | 32 | 2 | Voucher | Descriptions and perceptions related to a voucher system for BEP distribution |
| 43 | 30 | 1 | Healthy pregnancy | Anything about healthy pregnancy or healthy baby |
| 44 | 30 | 1 | Information Sources |  |
| 45 | 30 | 1 | Pregnancy Diet |  |
| 46 | 45 | 2 | Amount and/or frequency | anything about the amount of food or frequency of meals during pregnancy |
| 47 | 45 | 2 | Food classification | Mention of good or bad foods, organizing foods into categories, hot versus cold, etc. |
| 48 | 45 | 2 | Micronutrient supplements | Anything about micronutrient supplements |
| 49 | 45 | 2 | Special snacks or foods | anything about special snacks or foods in pregnancy |
| 50 | 30 | 1 | Screening | The methods that could be used or proposed for identifying beneficiaries, such as measuring weight or BMI. |
| 51 | 30 | 1 | Targeting | The identification of groups of potential beneficiaries, such as underweight women, certain SES groups, or other potential targets. |
| 52 | 30 | 1 | Technology |  |
| 53 | 30 | 1 | Weight gain | Anything about weight gain during pregnancy |

**COREQ (COnsolidated criteria for REporting Qualitative research) Checklist**

A checklist of items that should be included in reports of qualitative research. You must report the page number in your manuscript where you consider each of the items listed in this checklist. If you have not included this information, either revise your manuscript accordingly before submitting or note N/A.

| **Topic** | **Item No.** | **Guide Questions/Description** | **Reported on Page No.** |
| --- | --- | --- | --- |
| **Domain 1: Research team and reflexivity** |  |  |  |
| *Personal characteristics* |  |  |  |
| Interviewer/facilitator | 1 | Which author/s conducted the interview or focus group? | 6 |
| Credentials | 2 | What were the researcher’s credentials? E.g. PhD, MD | 6, 7 |
| Occupation | 3 | What was their occupation at the time of the study? | 6, 7 |
| Gender | 4 | Was the researcher male or female? | 7 |
| Experience and training | 5 | What experience or training did the researcher have? | 6,7 |
| *Relationship with participants* |  |  |  |
| Relationship established | 6 | Was a relationship established prior to study commencement? | 6, 11 |
| Participant knowledge of the interviewer | 7 | What did the participants know about the researcher? e.g. personal goals, reasons for doing the research | 6 |
| Interviewer characteristics | 8 | What characteristics were reported about the inter viewer/facilitator? e.g. Bias, assumptions, reasons and interests in the research topic | 6 |
| **Domain 2: Study design** |  |  |  |
| *Theoretical framework* |  |  |  |
| Methodological orientation and Theory | 9 | What methodological orientation was stated to underpin the study? e.g.  grounded theory, discourse analysis, ethnography, phenomenology, content analysis | 7 |
| *Participant selection* |  |  |  |
| Sampling | 10 | How were participants selected? e.g. purposive, convenience, consecutive, snowball | 6 |
| Method of approach | 11 | How were participants approached? e.g. face-to-face, telephone, mail, email | 6 |
| Sample size | 12 | How many participants were in the study? | 5 |
| Non-participation | 13 | How many people refused to participate or dropped out? Reasons? | n/a |
| *Setting* |  |  |  |
| Setting of data collection | 14 | Where was the data collected? e.g. home, clinic, workplace | 6 |
| Presence of nonparticipants | 15 | Was anyone else present besides the participants and researchers? | 6 |
| Description of sample | 16 | What are the important characteristics of the sample? e.g. demographic data, date | 5, 6, 8 |
| *Data collection* |  |  |  |
| Interview guide | 17 | Were questions, prompts, guides provided by the authors? Was it pilot tested? | 5, supp info |
| Repeat interviews | 18 | Were repeat inter views carried out? If yes, how many? | 5 |
| Audio/visual recording | 19 | Did the research use audio or visual recording to collect the data? | 6-7 |
| Field notes | 20 | Were field notes made during and/or after the inter view or focus group? | 6-7 |
| Duration | 21 | What was the duration of the inter views or focus group? | 6-7 |
| Data saturation | 22 | Was data saturation discussed? | n/a |
| Transcripts returned | 23 | Were transcripts returned to participants for comment and/or  correction? | n/a |
| **Domain 3: analysis and findings** |  |  |  |
| *Data analysis* |  |  |  |
| Number of data coders | 24 | How many data coders coded the data? | 7 |
| Description of the coding tree | 25 | Did authors provide a description of the coding tree? | 7, supp info |
| Derivation of themes | 26 | Were themes identified in advance or derived from the data? | 7 |
| Software | 27 | What software, if applicable, was used to manage the data? | 7 |
| Participant checking | 28 | Did participants provide feedback on the findings? | n/a |
| *Reporting* |  |  |  |
| Quotations presented | 29 | Were participant quotations presented to illustrate the themes/findings?  Was each quotation identified? e.g. participant number | 8-17 |
| Data and findings consistent | 30 | Was there consistency between the data presented and the findings? | 8-17 |
| Clarity of major themes | 31 | Were major themes clearly presented in the findings? | 8-17 |
| Clarity of minor themes | 32 | Is there a description of diverse cases or discussion of minor themes? | 8-17 |

Developed from: Tong A, Sainsbury P, Craig J. Consolidated criteria for reporting qualitative research (COREQ): a 32-item checklist for interviews and focus groups. *International Journal for Quality in Health Care*. 2007. Volume 19, Number 6: pp. 349 – 357

**Once you have completed this checklist, please save a copy and upload it as part of your submission. DO NOT** **include this checklist as part of the main manuscript document. It must be uploaded as a separate file.**
